# Intraoperative Radiation “Boost” to the Surgical Resection Bed following Pancreaticoduodenectomy for a Borderline Resectable Pancreatic Carcinoma: A Case Report

**DOI:** 10.3389/fonc.2018.00012

**Published:** 2018-03-23

**Authors:** Tarita O. Thomas, William Small, Mark Fleming, Song Kang, Richard A. Hoefer

**Affiliations:** ^1^Department of Radiation Oncology, Stritch School of Medicine, Loyola University, Chicago, IL, United States; ^2^Cardinal Bernardin Cancer Center, Maguire Center, Loyola University Medical Center, Maywood, IL, United States; ^3^Virginia Oncology Associates, Hampton, VA, United States; ^4^Division Chief Hematology and Oncology, Eastern Virginia Medical School, Norfolk, VA, United States; ^5^Department of Radiation Oncology, Sentara CarePlex Hospital, Sentara Healthcare, Hampton, VA, United States; ^6^Department of Surgical Oncology, Sentara CarePlex Hospital, Sentara Healthcare, Hampton, VA, United States; ^7^Eastern Virginia Medical School, Norfolk, VA, United States

**Keywords:** intraoperative radiation therapy, IORT, pancreas cancer, borderline resectable, neoadjuvant therapy

## Abstract

Neoadjuvant therapy including chemotherapy alone or concurrent chemotherapy with external bream radiation is a standard treatment strategy for borderline resectable pancreatic adenocarcinoma and is also used routinely for primary operable cancers at some institutions (1). The use of intraoperative radiation therapy (IORT) has been limited largely because of the logistical issues in delivery of radiation during surgery (2). This is the first reported case of a borderline resectable pancreas cancer patient who underwent neoadjuvant chemo-radiation therapy followed by resection with the use of IORT using the mobile IntraBeam device to boost the resection bed and improve local control by dose escalation.

## Introduction

Surgical resection remains the mainstay therapy for pancreas cancer; however, more than 80% of patients have disease that is not resectable at the time of diagnosis (3). There is a subgroup of patients who are considered potentially resectable following neoadjuvant therapy. This group has been termed borderline resectable pancreas cancer (BRPC) based on the assessment of the association of tumor with the major regional vessels. Generally, neoadjuvant therapy includes chemotherapy followed by concurrent chemo-radiation therapy or in some cases up front chemo-radiation therapy alone (4). Neoadjuvant chemo-radiation has been increasingly used for patients with localized pancreatic carcinoma including resectable or BRPC with improved outcomes (5, 6).

Retrospective studies have shown that adding local radiation to a BRPC leads to improvement in survival at 1 year due to improved local control (7). Phase II data and single institution data support the use of neoadjuvant therapy (8–10). In addition, a meta-analysis has shown that in about one-third of initially unresectable patients can be converted to resectable with the use of neoadjuvant therapy (7). Intraoperative radiation therapy (IORT) had been used in pancreas cancer in the past primarily with the goal of pain reduction and control of locoregional tumor progression (11–14). Formerly IORT was given with an electron energy source that produced promising results but was difficult to implement. Additionally, further radiation following neoadjuvant radiation after surgery for a close or positive margin is typically prohibited by normal tissue tolerances. Intraoperative radiation has been used as a method to deliver additional radiation boost to areas at risk for residual disease. Here, we describe the use of low-kilovoltage (low-kV) IORT to boost an area at risk for residual disease recognized during the surgical resection.

## Case Report

**Figure 1:**
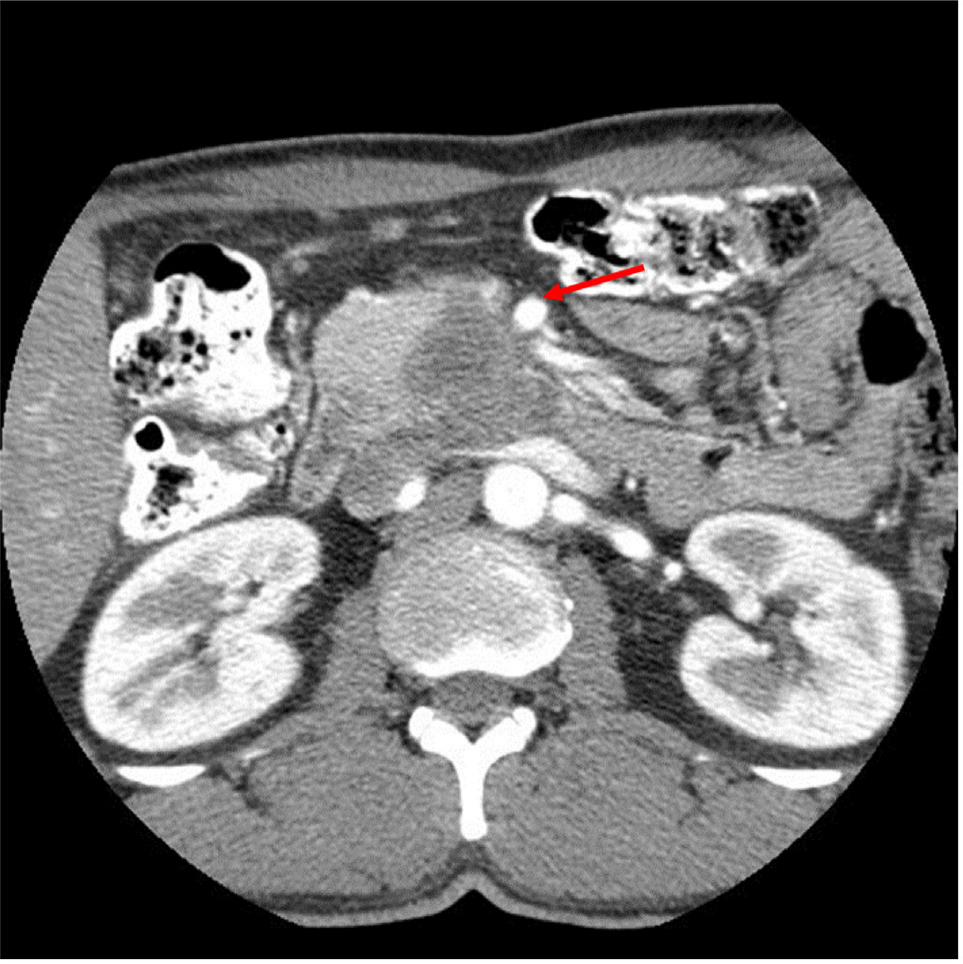
CT abdomen at initial work-up that shows a hypo-attenuated mass in the region of the uncinate process contacting 50% of the superior mesenteric vein, and approximately 25% of the superior mesenteric artery (see arrow) as well as contacting the left renal vein.

**Figure 2:**
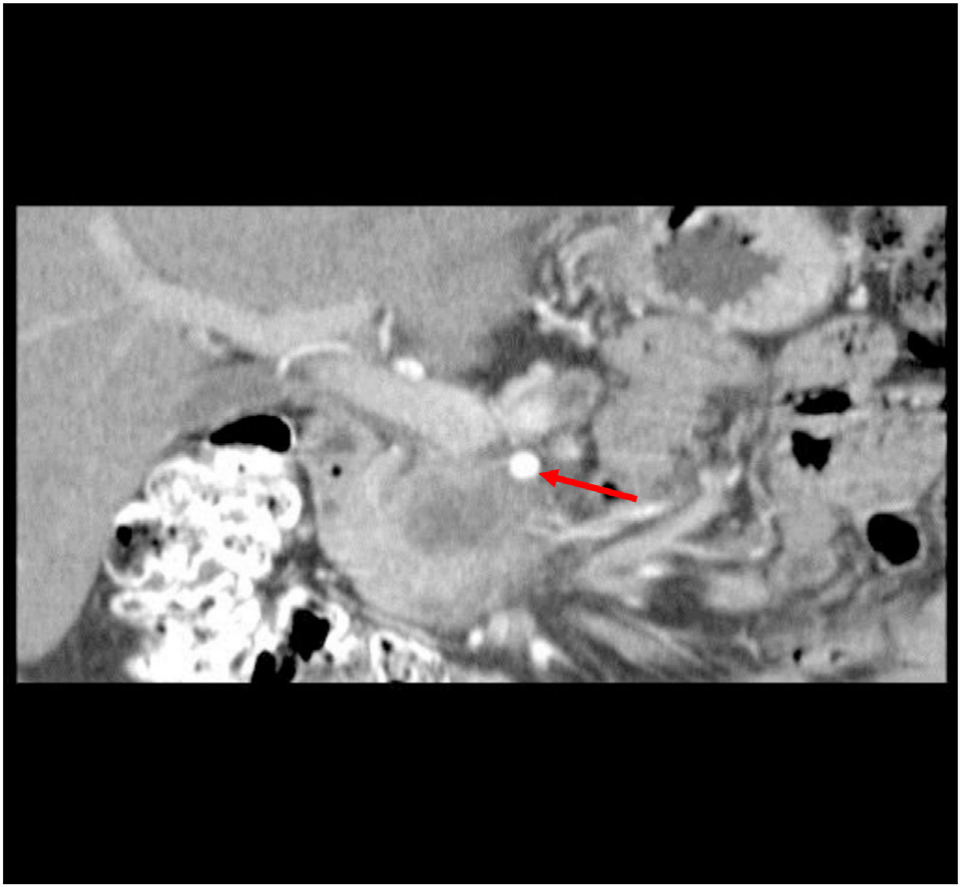
CT abdomen at restaging following neoadjuvant chemo-radiation therapy showed a 3.3 cm × 3.1 cm mass, decreased from initial size with involvement of the SMA (see arrow) and SMV improved.

**Figure 3:**
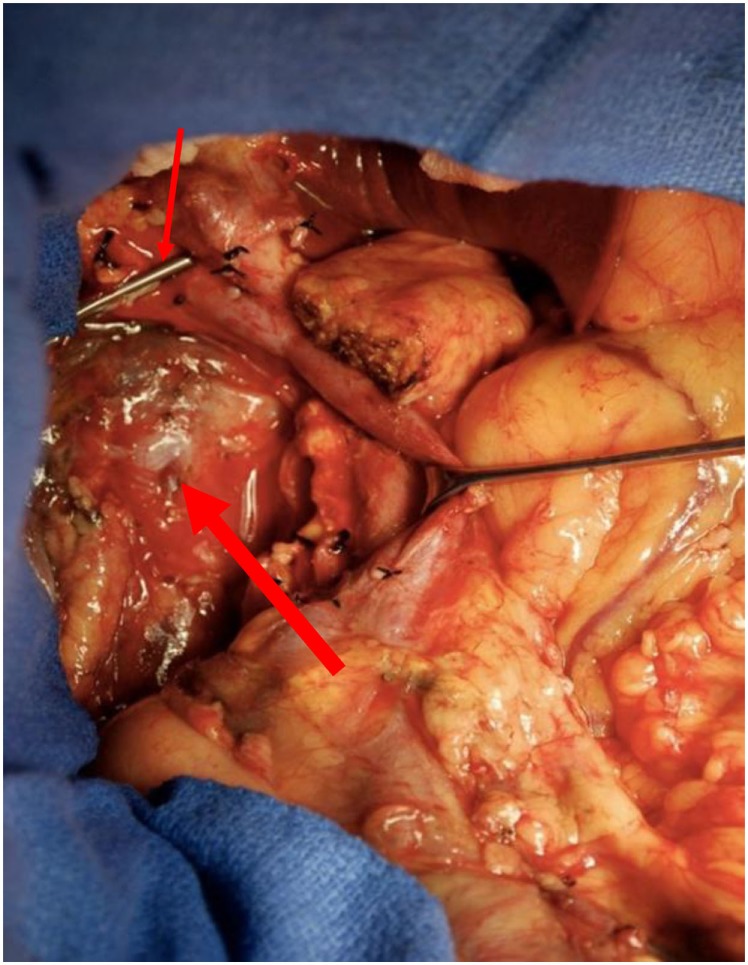
Surgical field at the time of pylorus-preserving pancreaticoduodenectomy in region of the uncinate margin at the superior mesenteric vein (thin arrow), superior mesenteric artery, and inferior vena cava were intraoperative low-kV radiation therapy was administered in retroperitoneal space (thick arrow).

A 50-year-old African-American male presented with a 14-day history of abdominal pain and anorexia in the spring of 2008. Abdominal CT scan revealed a 3.4 cm × 3.7 cm × 4.7 cm hypo-attenuated mass in the region of the uncinate process, contacting directly 50° of the superior mesenteric vein and approximately 25° of the superior mesenteric artery as well as contacting the left renal vein (Figure 1). The pancreatic duct was dilated measuring 0.7 cm. An endoscopic ultrasound-guided FNA biopsy was non-diagnostic, and percutaneous CT-guided biopsy confirmed a pancreatic carcinoma. CEA was 14.5 and Ca19-9 was 76. The case was discussed in prospective tumor board and the tumor was deemed to meet NCCN criteria for borderline resectable disease of less than or equal to 180° of contact of major vessels. The patient underwent neoadjuvant chemo-radiation with protracted infusion 5FU and involved field radiation to a dose of 5,040 cGy. Repeat staging showed a good response with the tumor measuring 3.3 cm × 3.1 cm, and the association of the tumor to the SMV and SMA being improved (Figure 2). Repeat Ca19-9 was 3. The patient was taken to surgery and underwent pylorus-preserving pancreaticoduodenectomy (Figure 3). A boost dose of 1,800 cGy was prescribed to the surface of a 3.5 cm spherical applicator and was administered intraoperatively to the uncinate margin at the superior mesenteric vein, superior mesenteric artery, and inferior vena cava. This area was felt to be at the highest risk for a close or positive margin and formed a concave region making it amendable for treatment with a spherical applicator. Normal tissue was retracted including the left kidney, neck of pancreas, small bowel, and colon which were protected with lead shields and several centimeters of tissue equivalent material in all directions other than the target. Once the radiation dose was delivered, the patient was reconstructed in the standard fashion. Pathology confirmed a 0.5 cm area of tumor over the inferior vena cava. Biopsies of the soft tissues along the right lateral border of the superior mesenteric vein were benign. The patient’s final pathology stage was ypT3, N0, M0 with 12 regional nodes negative for metastatic disease, and margins of resection were negative (R0). The patient had a satisfactory postoperative course and was discharged on the 11th postoperative day with the evidence of delayed gastric emptying requiring home TPN. The patient was, over time, able to return to a regular diet, and the TPN was discontinued and then received 6 months of adjuvant chemotherapy with gemcitabine. In our surgical series, we have found ~40% rate of some element of delayed gastric emptying. We have routinely placed patients with clinical manifestation of delayed gastric emptying in the first postoperative week on TPN. This has decreased readmission rates. In this case report, there was no need for vessel resection or reconstruction. This patient did undergo a pylorus-preserving procedure which may have a higher rate of delayed gastric emptying. In addition, it is possible that nerves in the retroperitoneal space could have had radiation exposure. The cause of this patient’s delayed gastric emptying is likely multi-factorial including the effects of the surgical technique and radiation exposure.

**Figure 4:**
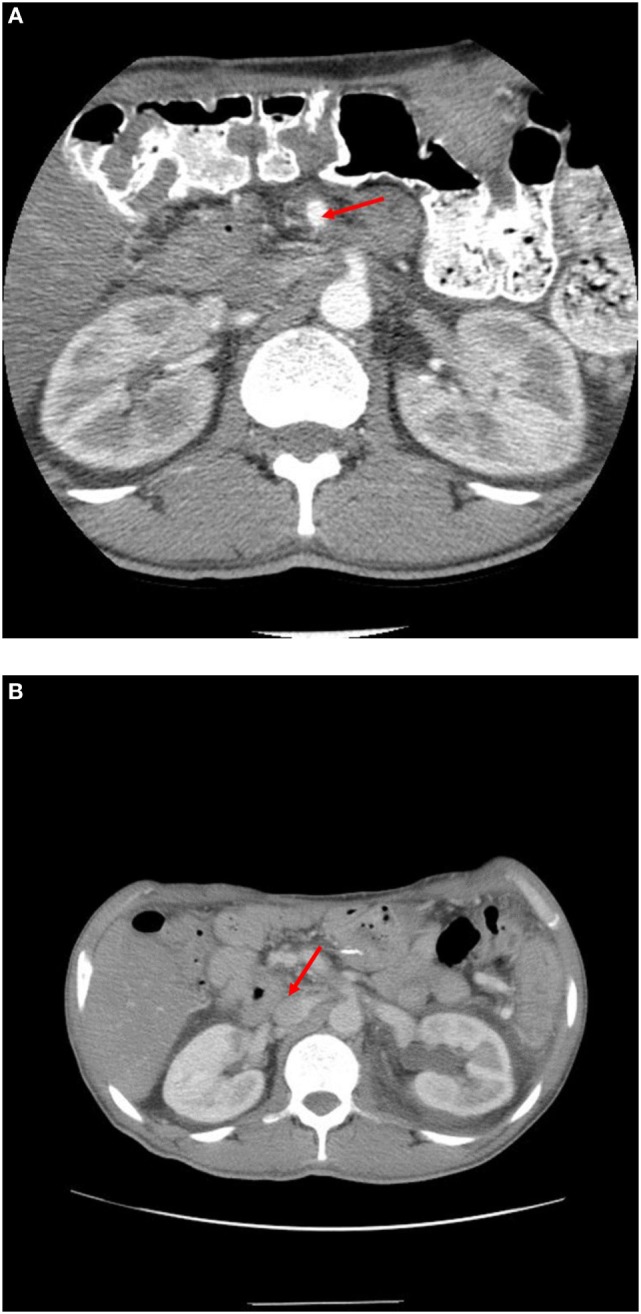
**(A)** 1 year post-operative scan showing SMA (see arrow) is clear of disease. **(B)** 7 year post-operative scan showing renal vein at IVC (see arrow) widely patient and without disease.

The patient has been followed with repeat three-dimensional imaging at routine intervals (Figure 4). Since surgery, the patient has been in surveillance follow-up for over 87 months and is considered free of disease.

## Discussion

Intraoperative radiation therapy refers to the delivery of a single dose of radiation therapy directly in the tumor bed at the time of surgery. IORT can be delivered with both electron and low-energy kV X-rays. IOERT requires a specially shielded and approved operating room with a linear accelerator or prior to closing the incision the patient is physically transported to a radiation oncology suite for treatment. In most institutions, this is prohibitive. In addition, electron therapy has more energy scatter and thus is more difficult to spare adjacent normal tissue compared to low-energy kV X-rays (15).

The Intrabeam system offers many potential advantages over IOERT including accessibility, easier patient positioning, rapid dose gradient, and beam characteristics. The system is portable allowing for use in multiple operating rooms. Therefore, no designated operating room is necessary for delivery of treatment. The system has six degrees of freedom permitting the machine to conform to multiple treatment positions. The low-kV X-rays have a steep dose gradient (15). The rapid dose gradient allows for delivery of high dose to the target tissue while relatively sparing the surrounding normal tissues (16). Any tissue at risk can be shielded with tungsten-filled silicone or even wet gauze. The system has minimal radiation protection requirements allowing essential staff to stay in the room with the anesthetized patient during delivery of the radiation.

In addition to treatment parameters that make the Intrabeam technology easier to deliver, there are also potential radiobiological benefits of IORT. Delivery of a single high-dose treatment to a confined area rather than a fractionated postoperative course of therapy does not allow for tumor cell proliferation between surgery and start of radiotherapy as well as sublethal damage repair between fractions in a standard course of radiation therapy (17, 18).

In summary, potential advantages of an intraoperative radiation include direct visualization of the tumor bed, no temporal delay between surgery and radiation not allowing for tumor cell repopulation, and improved displacement and shielding of normal tissues. Loyola University is accruing to a Phase I, 3 × 3 trial of 10, 15, and 20 Gy IORT prescribed to the surface of a flat applicator for patients with resectable pancreas cancer (19). This trial will further establish the dose of kV IORT to increase treatment to the tumor bed and retroperitoneal margin; the areas of highest risk of local recurrence. Currently, flat applicators are available in six sizes ranging from 10 to 60 mm to accommodate patient size and area of concern.

In BRPC, vascular involvement remains an important factor as to whether or not a patient can become resectable. Once respectable, the margin negative rate is a key determinant of local control. This case demonstrates the feasibility of using IORT for target dose escalation while minimizing the normal tissue exposure. This is the first known successful use of intraoperative radiation using the IntraBeam device for a BRPC patient.

## Ethics Statement

This study was carried out in accordance with the recommendations of Sentara CarePlex Hospital regulations with written informed consent from all subjects. All subjects gave written informed consent in accordance with the Declaration of Helsinki. The protocol was approved by the Sentara CarePlex Hospital committee on events related to human subjects.

## Author Contributions

TT and WS provided intellectual background in analyzing the data for this report and writing the report. MF, SK, and RH were directly involved in the treatment of the patient reported in this case report.

## Conflict of Interest Statement

WS is a member of US Targit trial Steering Committee and has received travel and speaker honorarium funds from Zeiss. RH and WS are board members of the TARGIT Collaborative Group. All other authors declare that the research was conducted in the absence of any commercial or financial relationships that could be construed as a potential conflict of interest.
